# Extraction of Explicit and Implicit Cause-Effect Relationships in Patient-Reported Diabetes-Related Tweets From 2017 to 2021: Deep Learning Approach

**DOI:** 10.2196/37201

**Published:** 2022-07-19

**Authors:** Adrian Ahne, Vivek Khetan, Xavier Tannier, Md Imbesat Hassan Rizvi, Thomas Czernichow, Francisco Orchard, Charline Bour, Andrew Fano, Guy Fagherazzi

**Affiliations:** 1 Center of Epidemiology and Population Health Inserm, Hospital Gustave Roussy Paris-Saclay University Villejuif France; 2 Epiconcept Company Paris France; 3 Accenture Labs San Francisco, CA United States; 4 Laboratoire d'Informatique Médicale et d'Ingénierie des Connaissances pour la e-Santé Inserm, University Sorbonne Paris Nord Sorbonne University Paris France; 5 Indian Institute of Science Bengaluru India; 6 Deep Digital Phenotyping Research Unit Department of Precision Health Luxembourg Institute of Health Strassen Luxembourg

**Keywords:** causality, deep learning, natural language processing, diabetes, social media, causal relation extraction, social media data, machine learning

## Abstract

**Background:**

Intervening in and preventing diabetes distress requires an understanding of its causes and, in particular, from a patient’s perspective. Social media data provide direct access to how patients see and understand their disease and consequently show the causes of diabetes distress.

**Objective:**

Leveraging machine learning methods, we aim to extract both explicit and implicit cause-effect relationships in patient-reported diabetes-related tweets and provide a methodology to better understand the opinions, feelings, and observations shared within the diabetes online community from a causality perspective.

**Methods:**

More than 30 million diabetes-related tweets in English were collected between April 2017 and January 2021. Deep learning and natural language processing methods were applied to focus on tweets with personal and emotional content. A cause-effect tweet data set was manually labeled and used to train (1) a fine-tuned BERTweet model to detect causal sentences containing a causal relation and (2) a conditional random field model with Bidirectional Encoder Representations from Transformers (BERT)-based features to extract possible cause-effect associations. Causes and effects were clustered in a semisupervised approach and visualized in an interactive cause-effect network.

**Results:**

Causal sentences were detected with a recall of 68% in an imbalanced data set. A conditional random field model with BERT-based features outperformed a fine-tuned BERT model for cause-effect detection with a macro recall of 68%. This led to 96,676 sentences with cause-effect relationships. “Diabetes” was identified as the central cluster followed by “death” and “insulin.” Insulin pricing–related causes were frequently associated with death.

**Conclusions:**

A novel methodology was developed to detect causal sentences and identify both explicit and implicit, single and multiword cause, and the corresponding effect, as expressed in diabetes-related tweets leveraging BERT-based architectures and visualized as cause-effect network. Extracting causal associations in real life, patient-reported outcomes in social media data provide a useful complementary source of information in diabetes research.

## Introduction

Diabetes distress refers to psychological factors such as emotional burden, worries, frustration, or stress in the day-to-day management of all types of diabetes [1-3]. Diabetes distress is associated with poor quality of life [4], high hemoglobin A_1C_ levels [5,6], and low medication adherence [7]. Reducing diabetes distress may improve hemoglobin A_1c_ levels and reduce the burden of disease among people with diabetes [8]. Social media is a useful observatory resource for patient-reported diabetes issues and could help to contribute directly to public and clinical decision-making from a patient’s perspective, given the active online diabetes community [9,10]. Identifying causal relations in expressed text data in social media platforms might help to discover unknown etiological results, specifically, causes of health problems, concerns, and symptoms.

To intervene and potentially prevent diabetes distress, it is necessary to understand the causes of diabetes distress from a patient’s perspective to understand how patients see their disease. Causal relation extraction in natural language text has gained popularity in clinical decision-making, biomedical knowledge discovery, or emergency management [11]. In particular, causal relations on Twitter have been examined for diverse factors causing stress and relaxation [12], adverse drug reactions [13], or causal associations related to insomnia or headache [14]. Most approaches examine *explicit* causality in text [14-16], when cause and effect are explicitly stated, for instance, by connective words (eg, so, hence, because, lead to, since, if-then) [11,17]. An example for an *explicit* cause-effect pair is “diabetes causes hypoglycemia.” However, *implicit* causality is more complicated to detect such as in “I reversed diabetes with lifestyle changes” with cause “lifestyle changes” and effect “reversed diabetes.”

Natural language processing methods explore among other things how computers can be used to extract useful information from natural language documents. In combination with machine learning and deep learning models, which are artificial intelligence algorithms designed to learn from experience, they have also been applied to extract causal relations [18,19]. Machine learning methods are able to explore implicit relations and provide better generalization contrary to rule-based approaches [11,20-22]. An interesting approach leveraging the transfer learning paradigm and addressing both explicit and implicit cause-effect extraction is provided by Khetan et al [23]. They fine-tuned pretrained transformer-based Bidirectional Encoder Representations from Transformers (BERT) language models [24,25] to detect “cause-effect” relationships by using publicly available data sets such as the adverse drug effect data set [26]. More generally, the idea of transfer learning is to leverage the knowledge of a model that has been trained on an auxiliary domain [27].

In this study, we aimed to extract spans of text as 2 distinct events from diabetes and diabetes-related tweets such that one event directly (explicit) or indirectly (implicit) impacts another event. We categorized these events as cause-event and effect-event depending upon the expressed context of each tweet. The identified cause and effect will then be aggregated into clusters and ultimately visualized in an interactive cause-effect network.

This work is realized in the frame of the World Diabetes Distress Study, which aims to analyze what is shared on social media worldwide to better understand what people with diabetes and diabetes distress are experiencing [28,29]. The social network “Twitter” is a popular data resource among diabetes researchers owing to its public character and its active online diabetes community compared to other social media [30,31]. Recent studies suggest an overrepresentation of people with type 1 diabetes compared to those with type 2 diabetes who are active on Twitter [9,31].

## Methods

### Overview

**Figure 1:**
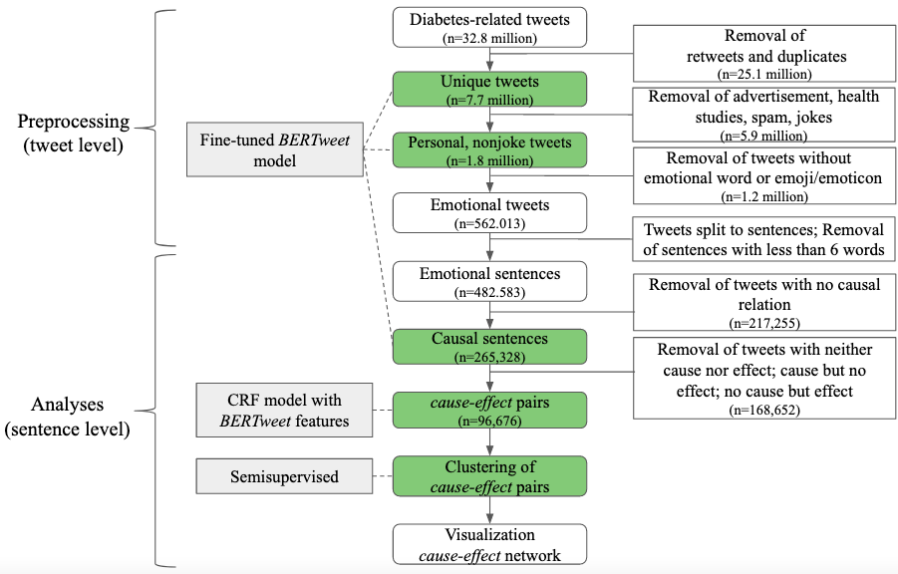
Workflow. The steps shown in green include machine learning methods. CRF: conditional random field.

On the basis of diabetes-related tweets, we first preprocessed tweets to only focus on personal, nonjoke, and emotional content. Second, after this preprocessing step, we split tweets into sentences for our analyses, as we aimed to identify the cause-effect relationships between events within a sentence (sentence level) and not across multiple sentences (tweet level). This also simplifies model training and helps with easier learning. Third, we identified sentences in which causal information (opinion, observation, etc) is communicated. In the fourth step, causes and their corresponding effects were extracted. Lastly, those cause-effect pairs were aggregated, described, and visualized. The entire workflow is illustrated in Figure 1.

### Data Collection and Ethical Considerations

Via Twitter’s streaming application programming interface, 32 million diabetes-related tweets in English were collected between April 2017 and January 2021 based on a list of diabetes-related keywords such as *diabetes, hypoglycemia, hyperglycemia,* and *insulin* from all over the world (see Multimedia Appendix 1 for the full list of keywords used). This is an extended data set of the one used in earlier works [9]. All data collected in this study were publicly posted on Twitter. Therefore, according to the privacy policy of Twitter, users agree to have this information available to the general public [30].

### Data Preprocessing

Tweets are noisy and unstructured. They contain many misspelled or nonstandard English words. To reduce noise in the data set, we applied a preprocessing pipeline similar to that in earlier works, the details of which are summarized in Figure 1 [9]. First, retweets and duplicates were removed to obtain a database with 7.7 million unique tweets. Second, we determined only tweets with *personal* content where feelings, emotions, and opinions could be shared by people with or talking about diabetes and excluded *institutional* tweets referring to commercial, news, or health information. To identify *personal* content in tweets, we leveraged the transfer learning paradigm and fine-tuned the already pretrained transformer-based language model *BERTweet*, which was pretrained on 850 million English tweets (16 billion word tokens ~ 80 GB) [25,32]. To use the model and fine-tune it for a binary sentence classification, a linear layer was added on top of the last transformer layer of the *BERTweet* model by using the *transformers* package of HuggingFace [33]. The model was then fine-tuned with an extended data set of one used in earlier works, leading to a total of 4303 tweets (1539 *personal* and 2764 *institutional*) to account for a possible temporal divergence of the way people tweet [9]. The model performance to identify tweets with personal content had accuracy of 91.2%, precision of 86.2%, recall of 90.9%, and F1 score of 88.5%. The trained model was then applied to all unique tweets, resulting in a total of 2.5 million tweets with personal content. Moreover, jokes around diabetes are common on Twitter and were considered out of scope for this study as well. Similar to the *personal* content classifier, *BERTweet* was fine-tuned to detect if a tweet is a joke. For this purpose, a joke tweet data set from earlier works was extended to 1648 tweets (486 jokes, 1162 nonjokes) [9]. The performance to identify if a tweet is a joke had accuracy of 90.4%, precision of 78.5%, recall of 90.8%, and F1 score of 84.2%. Applying the joke classifier on all tweets with personal content led to a data set of 1.8 million personal nonjoke tweets.

A particular focus of this study was on studying diabetes distress and thus, the psychological factors and emotions. To capture these factors in tweets, only tweets containing an emotional element such as emojis/emoticons or emotional words were kept. Emotional words were identified based on a combination of the psychologue Parrot’s hierarchical classification of emotions with the 6 primary emotions (*joy, love, surprise, sadness, anger, fear*) and emotional words present in common questionnaires to study diabetes distress such as the Problem Areas in Diabetes scale and Diabetes Distress Scale [34-36]. This led to 562,013 tweets containing personal, nonjoke, and emotional content. More details on the preprocessing pipeline are summarized in Multimedia Appendix 2 [9,25,32-40].

### Data Annotation

In order to identify causal sentences and *cause-effect* association, 5000 randomly chosen diabetes-related tweets were selected, preprocessed, split into sentences, and then manually labeled. We did not restrict ourselves to a specific area of diabetes-related causal relationships, and we included potentially all types. Table 1 illustrates some example sentences. Only causal relationships related to diabetes were labeled as positive samples, whereas non–diabetes-related or unclear cause-effect relationships were labeled as negative samples. For a more detailed explanation on the annotation, please refer to our annotation guidelines in Multimedia Appendix 3.

**Table 1:** Sample sentences in different label scenarios. The examples are fictive to ensure privacy.

Sentences	Cause	Effect	Causal association	Explanation
Diabetes causes me to have mood swings	Diabetes	mood swings	1	Possible causal association
I just want to eat, I hate #diabetes	#diabetes	hate	1	Possible causal association related to diabetes distress
Scary, have a diabetic daughter but I read thousands of people a year die in the United Kingdom just from flu so why panic over corona.	—^a^	—	0	Nondiabetes or diabetes distress–related relationship. “Flu” is not diabetes-related
Had two strokes and recover now and also have high blood pressure and diabetes. 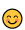	—	—	0	Unclear cause-effect relationship. Not clear if “high blood pressure” or “diabetes” caused the stroke
Not sure if I've been up since 3:30 to watch Titanic or because of my anxiety over my glucose test is what keeps me up 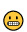	glucose test	anxiety	1	Chaining cause-effect relationship (A->B->C) Event A: glucose test Event B: anxiety Event C: been up since 3:30 => label the relationship which is closest to our study objective: diabetes and diabetes distress
My 14-year-old daughter is type 1 = malfunctioning pancreas, meaning not enough insulin being made to regulate 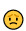	type 1	malfunctioning pancreas; not enough insulin	1	Negation in a cause/effect is considered being part of the cause/effect as it does not alter the meaning
It is not true to think that insulin makes you feel so bad 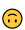	insulin	feel so bad	0	Negation is not part of cause/effect and alters the meaning

^a^Not available.

Labeling cause-effect pairs is a complex task. To verify the reliability of the labeling, 2 authors labeled 500 sentences independently and we calculated Cohen κ score, a statistical measure expressing the level of agreement between 2 annotators [41]. We obtained a score of 0.83, which is interpreted as an *almost perfect* agreement according to Altman [42] and Landis and Koch [43]. Disagreements were discussed between 2 authors, and 1 author labelled the other samples, resulting in 8235 labelled sentences (7218 noncausal sentences and 1017 causal sentences) from 5000 tweets.

### Models

The first model was trained to predict if a sentence contains a potential cause-effect association (causal sentence), and the second model extracted the specific cause and the associated effect from the causal sentence. Thus, the first model acts like a barrier and filters noncausal sentences out. These sentences may have either a cause, an effect, none of them, but not both. To simplify the model training, we hypothesized that cause-effect pairs only occur in the same sentence and we removed all sentences with less than 6 words owing to a lack of context. For this reason, we operated on a sentence level and not at the tweet level. Additional challenges in our setting were that *causes* and *effects* could be multiword entities and the language used on Twitter is nonstandard with frequent slang and misspelled words.

### Causal Sentence Detection

**Figure 2:**
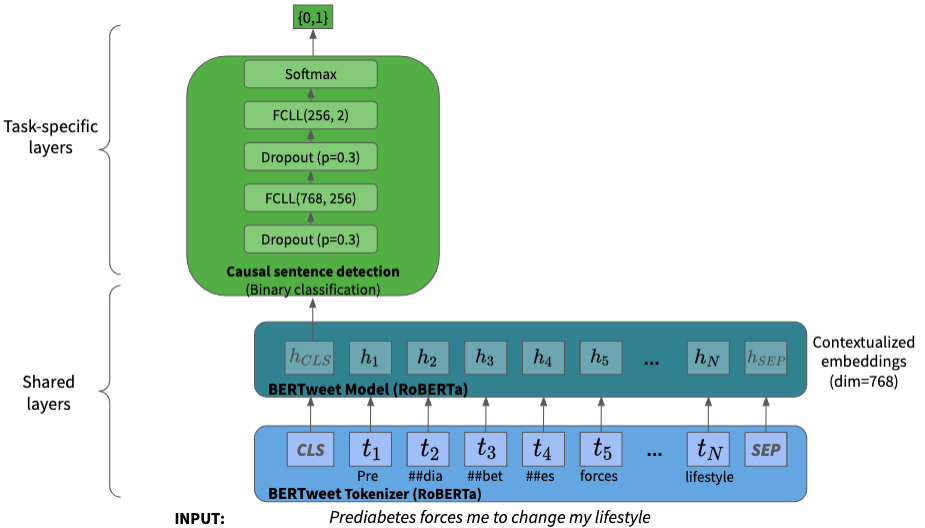
Model architecture for causal sentence detection. FCLL: fully connected linear layer; p: probability of an element to be zeroed.

The identification of causal sentences is a binary classification task. The pretrained language model *BERTweet* served as a foundation for the model architecture capable of handling the nonstandard nature of Twitter data [32]. A feed-forward network is built on top of the *BERTweet* [32] architecture consisting of 2 fully connected layers with dropout layers with a probability of 0.3, finalized by a softmax layer, which translates the model predictions into probabilities (Figure 2). To adjust for the class imbalance in the labeled data, class weights were included as parameters in the categorical cross-entropy loss function to penalize mispredictions for causal sentences strongly. Initially, labelled data were stratified, and 10% of it was kept as test set. The remaining 90% of the samples were further separated into training and validation sets with 80:20 split.

### Data Augmentation Through Active Learning

**Figure 3:**
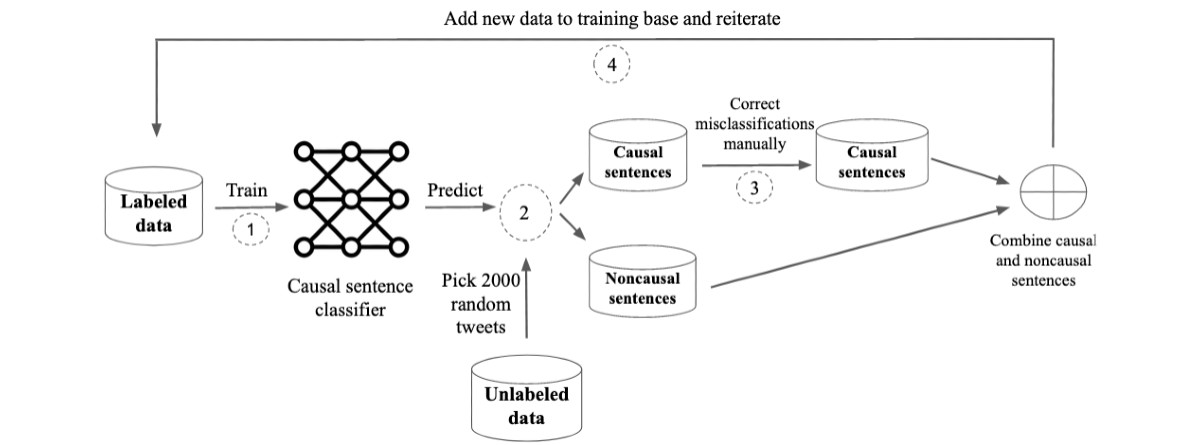
Active learning loop to augment the training set in a time-efficient fashion.

Data imbalance on the one hand and the limited number of positive training examples for each cause-effect pair on the other hand (as causes and effects could potentially be related to any concept in the diabetes domain) drove us to adopt an active learning approach to increase the training data. Active learning is a sample selection approach aiming to minimize the annotation cost while maximizing the performance of machine learning–based models [44]. It has been widely applied on textual data [45,46]. The training data were increased in several iterations, as illustrated in Figure 3.

The first iteration started by training the causal sentence classifier on sentences from the 5000 tweets. The trained classifier was then applied on 2000 randomly selected unlabeled tweets, which were preprocessed and split into sentences, resulting in a set of causal sentences and a set of noncausal sentences. The sentences predicted as causal sentences were examined manually, and possible misclassifications were corrected to ensure clean positive training samples. The noncausal sentence set remained untouched. As a consequence, potential misclassifications remained in the noncausal sentence set, which should then be considered noisy. Both the causal and noncausal sentence set were then combined and added as new training data to the already labeled data, leading to an updated training set of 7000 tweets. This process was iterated 4 times and allowed us to augment the labelled data much faster and more efficiently than that without active learning, as it enables us to focus on the few positive samples. The final training set was used to train the classification model and the cause-effect extraction model.

### Cause-Effect Pairs

After having trained the causal sentence classifier to detect sentences with causal information, we identified the specific cause-effect pairs in the causal sentences. The identification of cause-effect pairs was casted as an event extraction or named-entity recognition task, that is, assigning a label cause or effect to a sequence of words. The manually labeled causes and effects were encoded in an IO tagging format based on the common tagging format BIO (Beginning, Inside, Outside), introduced by Ramshaw and Marcus [47]. Here, “I-C” denotes inside the cause and “I-E” inside the effect. Those 2 tags were completed by the outside tag “O,” symbolizing that the word is neither cause nor effect. The IO tagging scheme for the example sentence with cause “prediabetes” and effect “change my lifestyle” is summarized:

Sentence: Prediabetes, forces, me, to, change, my, lifestyle

IO tags: I-C, O, O, O, I-E, I-E, I-E

**Figure 4:**
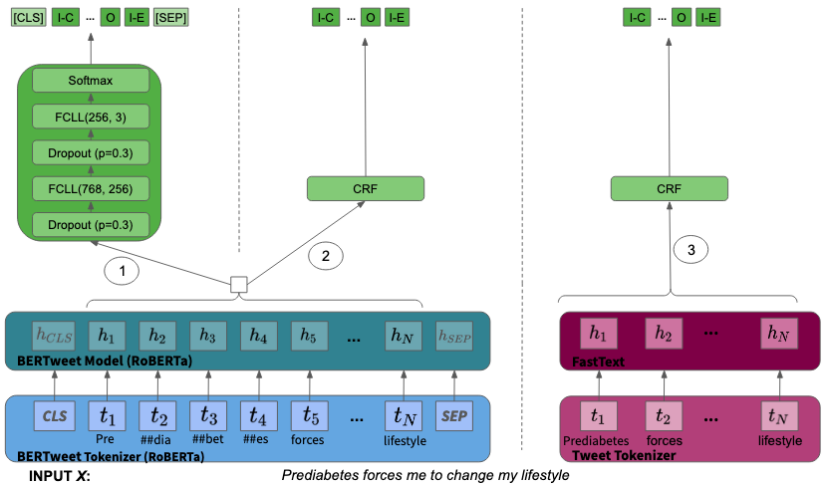
Model architectures of cause-effect identification. CRF: conditional random field; FCLL: fully connected linear layer; p: probability of an element to be zeroed.

Note that a word can be both cause or effect depending on the context. For instance “prediabetes” in “Prediabetes forces me to change my lifestyle” takes the role of a cause, whereas in “Limited exercising may lead to prediabetes,” it is a possible effect. IO tagging was preferred over BIO tagging to simplify the model learning by reducing the number of class from 5 to 3. Moreover, the task is complex and considered open domain, as causes and effects are not restricted to 1 specific topic but can be related to any concept in our target domain (diabetes). As a consequence, the creation of a representative training set is challenging, as most cause-effect pairs occur rarely. This complexity drove us to test several model architectures; refer to Figure 4 for an overview.

1. BERT_FFL: Pretrained BERTweet language model and on top, 2 feed forward layers with a dropout of 0.3, followed by a softmax layer. For the model training, the cross-entropy loss function is selected and weighted by the class weights to penalize mispredictions for causes and effects stronger.
2. WE_BERT_CRF: Single conditional random field (CRF) layer with BERTweet embeddings as features augmented by discrete features such as if the word is lowercase, digit, or the word length. CRFs are a standard statistical sequential classification method to identify entities in a text [48]. The CRF function is implemented with the python package sklearn-crfsuite [49] based on CRFsuite [50]. As parameters for the CRF function, the default algorithm “Gradient descent using the Limited Memory Broyden-Fletcher-GoldfarbShanno method” was chosen, and the coefficient for L1 and L2 regularization was 0.1.
3. FastText_CRF: Similar to WE_BERT_CRF, with the difference that BERTweet embeddings were replaced by FastText embeddings in the feature vector for each word. FastText vectors trained on similar diabetes-related tweets, which were well adapted to our use case [9].

### Clustering of Causes and Effects

A large part of *causes* and *effects* can be regrouped into similar concepts (clusters) to facilitate analyses and allow effective network analyses. We chose a semisupervised, time-efficient approach in which 1000 *causes* and 1000 *effects* were randomly chosen and 2 researchers manually grouped these into clusters such as “diabetes,” “death,” “family,” and “fear,” hereinafter referred to as “parent clusters” to simplify understanding. The remaining *causes* and *effects* were then automatically compared to each element of all the clusters based on *FastText* vectors and cosine similarity and associated with the cluster containing the most similar element. Experimentally, a similarity threshold of 0.55 was determined; if a cause/effect had a similarity smaller than this threshold for all elements, a new cluster was created for this cause/effect. These clusters were also visualized in an interactive cause-effect network, developed in D3, to enable further exploration of the cause-effect association about diabetes distress communication in social media. Python (version 3.8.8) and the deep learning framework PyTorch (version 1.8.1) were used to implement the abovementioned methods. The algorithms are open sourced under [51].

## Results

The following results were obtained from 482,583 sentences, which were obtained from splitting the 562,013 personal, emotional, and nonjoke tweets into sentences, excluding questions and including only sentences with more than 5 words.

### Model Training and Performance

#### Causal Sentences

Hyperparameters for the model training were optimized, and the best model was trained with an Adam optimizer with a learning rate of 1e-3 among [1e-2, 1e-3, 1e-4] and a scheduler with linearly decreasing learning rate with 0 warmup steps. The optimal batch size was obtained for 16 among [8,16,32], and we trained for 35 epochs with early stopping. The performances to detect causal sentences for the imbalanced data set are illustrated in Table 2 for each round of the active learning loop, with each round having been trained on more data. The highest accuracy was reached in round 4 with 71%. We applied the model of round 4 on all the remaining tweets, as it was trained on the largest training data set, including difficult causal examples missed by earlier models and is thus better at identifying complex causal sentences. The active learning strategy led us to increase the training data much quicker than that without active learning and without loss in performance. This led to a clean database of 265,328 causal sentences with the most noisy sentences removed.

**Table 2:** Performance measures (macro) for each round of more training data.

Round	Sentences in training set (n)	Sentences in test set (n)	Accuracy (%)	Precision (%)	Recall (%)
0	6024	837	64.5	58.0	67.4
1	7536	1047	67.7	61.2	71.6
2	8804	1223	67.7	60.3	66.3
3	10,284	1429	65.4	60.0	68.8
4	11,861	1648	71.0	61.0	67.8

#### Cause and Effect Detection

After having identified the causal sentences, the cause-effect models were trained to extract the specific cause-effect pairs. The active learning strategy led to an extended data set of 2118 causal sentences, that is, containing both cause and effect, of which 10% were used as a test set while the remaining 90% were further used to create a training and validation set with an 80:20 split. The performances of the different cause-effect models are listed in Table 3. The best performing model was the CRF model with BERT-embedding features (WE_BERT_CRF) with a precision, recall, and F1 score of 0.68. Surprisingly, it outperforms fine-tuning a BERT model, which is considered the gold standard of current named-entity recognition tasks. A potential explanation for this is that BERT-based models make local decisions at every point of the sequence taking the neighboring words into account before its decision. In a situation like ours, with strong uncertainty on all elements, owing to the complexity of the task, a single CRF layer model leveraging BERT features, making global decisions using the local context of each word, maximizes the probability of the whole sequence of the decision better. Moreover, the CRF model with simpler FastText models achieved strong results as well with one reason being probably that the word embeddings were specifically trained on this diabetes corpus.

Consequently, the WE_BERT_CRF model was applied on all causal sentences leading to a data set of 96,676 sentences with the *cause* and associated *effect* predicted.

**Table 3:** Performance measures for each of the 4 architectures.

Models			Precision		Recall		F1 score
**BERT_FFL**							
	I-C	0.48		0.46		0.47	
	I-E	0.20		0.48		0.29	
	O	0.91		0.77		0.83	
	macro	0.53		0.57		0.53	
**WE_BERT_CRF**							
	I-C	0.63		0.61		0.62	
	I-E	0.49		0.49		0.49	
	O	0.93		0.93		0.93	
	macro	0.68		0.68		0.68	
**FastText_CRF**							
	I-C	0.59		0.57		0.58	
	I-E	0.45		0.38		0.41	
	O	0.92		0.94		0.93	
	macro	0.65		0.63		0.64	

###  Cause-Effect Description

The semisupervised clustering led to 1751 clusters. To remove noisy clusters through potential misclassifications, only clusters with a minimal number of 10 cause/effect occurrences were considered for the following analyses, resulting in 763 clusters. Note that the order of documents might affect the results, as different clusters might have been created. Please refer to Multimedia Appendix 4 for an overview over the 100 largest clusters (automatically added clusters have “other” as “parent cluster”).

Table 4 provides an overview over the largest clusters, containing either cause or effect. Table 5 provides the most frequent cause-effect associations, excluding the largest cluster “diabetes,” as it will be studied separately. The cluster “diabetes” is the largest one with 66,775 occurrences of “diabetes” as either cause or effect (eg, diabetes, #diabetes, diabetes mellitus) followed by “death” with 16,989 (eg, passed away, killed, died, suicide) and “insulin” (eg, insulin, insulin hormone) with 14,148 occurrences. From the 30 largest clusters, 6 refer to nutrition, 4 to diabetes, and 3 to each of insulin, emotions, and the health care system. The most frequent cause-effect is “unable to afford insulin,” which causes “death” expressed in 1246 cases, followed by “insulin” causing “death” with 1156 cases and “type 1 diabetes” causing “fear” with 1054 cases.

The largest cluster “diabetes” mainly occurs as a cause and its 10 most frequent effects are death (n=7446), fear (n=4836), sick (n=2799), neuropathy (n=2477), hypoglycemia (n=2062), anger (n=1908), suffer (n=1808), insulin (n=1605), overweight (n=1506), and reduce weight (n=1487). From the 30 most numerous effects for “diabetes,” 6 were related to “nutrition” and 5 to “complications and comorbidities” and 3 to each of “diabetes distress,” “emotions,” and “health care system.”

**Figure 5:**
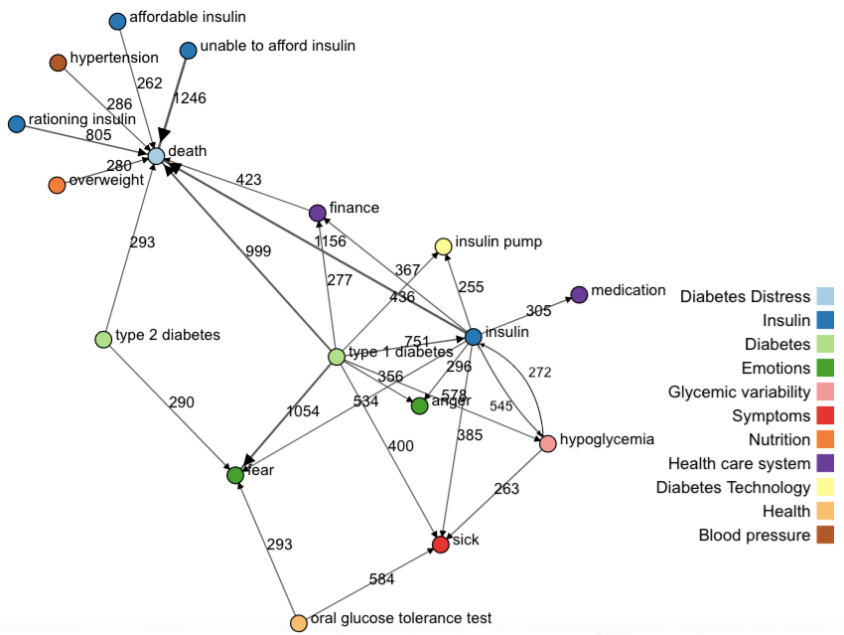
Cause-effect network with a minimum number of associations (edges) of 250. Accessible in [52].

The interactive visualization in D3 with filter options is published in [52]. Figure 5 provides an example graph of this visualization showing only cause-effect relationships with at least 250 occurrences to ensure readability. It is striking that “death” seems to play such a central role as *effect* with various causes (unable to afford insulin, rationing insulin, finance, insulin, type 1 diabetes, overweight) pointing at it. Other central nodes are type 1 diabetes acting as cause for insulin pump, insulin, hypoglycemia (hypo), sickness, finance, and anger, and fear emotions, where the latter has the strongest association, or the node “insulin” mostly relating as cause for sickness, medication, finance, death, or hypoglycemia and fear and anger.

**Table 4:** The most frequent clusters (causes and effects) with the number of occurrences.

Parent cluster	Cluster	Value (n)
Diabetes	diabetes	66,775
Death	death	16,989
Insulin	insulin	14,148
Diabetes	type 1 diabetes	11,693
Emotions	fear	10,160
Glycemic variability	hypoglycemia	9547
Symptoms	sick	6549
Nutrition	overweight	5186
Diabetes	type 2 diabetes	4909
Complications and comorbidities	neuropathy	4481
Health care system	medication	4389
Diabetes Technology	insulin pump	4307
Nutrition	nutrition	4230
Emotions	anger	4149
Health	oral glucose tolerance test	4053
Blood pressure	hypertension	3782
Health care system	finance	3767
Nutrition	reduce weight	3589
Insulin	unable to afford insulin	3381
Nutrition	diet	3325
Emotions	sadness	3153
Glycemic variability	hyperglycemia	3144
Diabetes	suffer	3132
Diabetes Distress	depression	2810
Health care system	hospital	2721
Diabetes Distress	stress	2681
Nutrition	sugar	2369
Nutrition	fasting	2363
Insulin	rationing insulin	2244
Health	gestational diabetes	2076

**Table 5:** The most frequent cause-effect relationships excluding the cluster “diabetes” with the number of occurrences.

Cause	Effect	Value (n)
unable to afford insulin	death	1246
insulin	death	1156
type 1 diabetes	fear	1054
type 1 diabetes	death	999
rationing insulin	death	805
type 1 diabetes	insulin	751
oral glucose tolerance test	sick	584
type 1 diabetes	hypoglycemia	578
insulin	hypo	545
insulin	fear	534
type 1 diabetes	insulin pump	436
finance	death	423
type 1 diabetes	sick	400
insulin	sick	385
insulin	finance	367
type 1 diabetes	anger	356
insulin	medication	305
insulin	anger	296
oral glucose tolerance test	fear	293
type 2 diabetes	death	293
type 2 diabetes	fear	290
hypertension	death	286
overweight	death	280
type 1 diabetes	finance	277
hypoglycemia	insulin	272
hypoglycemia	sick	263
affordable insulin	death	262
insulin	insulin pump	255
complications	death	248
insulin	sadness	240

## Discussion

### Principal Findings

Our findings suggest that it is feasible to extract both explicit and implicit causes and associated effects from diabetes-related Twitter data. We demonstrated that by adopting the transfer learning paradigm and fine-tuning a pretrained language model, we were able to detect causal sentences. Moreover, we have shown that simply fine-tuning a BERT-based model does not always outperform more traditional methods such as relying on CRFs in the case of the cause-effect pair detection. The precision, recall, and F1 scores, given the challenging task and the imbalanced data set, were satisfying. The semisupervised clustering and interactive visualization enabled us to identify “diabetes” as the largest cluster acting mainly as the cause for “death” and “fear.” Besides, a central cluster was detected in “death” acting as an effect for various causes related to insulin pricing—a link that was already detected in earlier works [9]. From a patient’s perspective, we were able to show that their main fear is insulin pricing, which is expressed in the most frequent cause-effect relationship “unable to afford insulin” causing “death” or “rationing insulin” causing “death.” As the main diabetes distress–related causes, we identified fear of hypoglycemia, insulin, hypertension, or the oral glucose tolerance test.

### Comparison With Previous Works

Several former works have addressed causality on Twitter data. Doan et al [14] focused on 3 health-related concepts, namely, stress, insomnia, and headache as effects and identified causes by using manually crafted patterns and rules. However, they only focused on explicit causality and excluded causes and effects encoded in hashtags and synonymous expressions [14]. On the contrary, we tackled both explicit and implicit causality, including causes and effects in hashtags and exploiting synonymous expressions through the use of word embeddings. Kayesh et al [16] proposed an innovative approach, a novel technique based on neural networks, which uses common sense background knowledge to enhance the feature set, but they focused on the simplified version of explicit causality in tweets. Bollegala et al [53] developed a causality-sensitive approach for detecting adverse drug reactions from social media by using lexical patterns and thereby aiming at explicit causality. Dasgupta et al [54] proposed one of the few deep learning approaches due to the unavailability of appropriate training data, leveraging a recursive neural network architecture to detect cause-effect relations from text, but they also only targeted explicit causality. A BERT-based approach tackling both explicit and implicit causality is provided by Khetan et al [23] who used already existing labeled corpora not based on social media data. Recently, they further extended their work of explicit and implicit causality understanding in single and multiple sentences but in clinical notes [55]. To the best of our knowledge, this is the first paper investigating both explicit and implicit cause-effect relationships on diabetes-related Twitter data.

### Strengths and Limitations

This study demonstrates various strengths. First, by leveraging powerful language models, we were able to identify a large number of tweets containing *cause-effect* relationships, which enabled us to the detect cause-effect associations in 20% (96,676/482,583) of the sentences, contrary to other approaches that were able to identify causality in less than 2% of tweets [14]. Second, contrary to most previous work, we tackled both explicit and implicit *causal relationships*, an additional explanation for the higher number of *cause-effect* associations we obtained, compared to other studies focusing only on explicit associations [14]. Third, relying fully on automatic machine learning algorithms avoided us from defining manually crafted patterns to detect causal associations. Fourth, operating on social media data that are expressed spontaneously and in real time offers the opportunity to gain knowledge from an alternative data source and, in particular, from a patient’s perspective, which might complement traditional epidemiological data sources. Lastly, the data-driven approach to identify cause-effect relationships, as reported from Twitter users, can be used in the next step to generate new hypotheses that can be tested in a more clinical setting, for example, in a clinical trial.

A strong limitation is that *cause-effect* relations are expressed in tweets and this cannot be used for causal inference as the Twitter data source is uncertain and the information shared can be an opinion or an observation. Another shortcoming is that the performance of our algorithms to detect *cause-effect* pairs is not perfect. However, the overall process and the vast amount of data minimize this issue. The lack of recall is counterbalanced by the sheer amount of data, and the lack of precision is counterbalanced by the clustering approach in which nonfrequent causes or effects are discarded [56]. Labeling causes and effects in a data set is a highly complicated task, and we would like to emphasize that mislabeling in the data set may occur. Here, the actual prevalence of causal sentences is lower, as we wanted to catch as many causal sentences as possible, which led to also having captured some noncausal sentences. Enhancing data quality certainly is a strong point to address to further improve performance. The causal association structures learnt by the model from the training set might not generalize completely when applied on the large amount of Twitter data. Besides, the active learning strategy certainly added noise to the model, as only positive samples were corrected, which could be improved in future investigations. Moreover, we would like to highlight that the diabetes-related information shared on Twitter may not be representative for all people with diabetes. For instance, we observed a bigger cluster of causes/effects related to type 1 diabetes compared to that related to type 2 diabetes, which is contrary to that in the real world [57]. A potential explanation for that is the age distribution of Twitter users [58]. However, owing to the large number of tweets analyzed, a significant variability in the tweets could be observed.

### Conclusion

In this work, we developed an innovative methodology to identify possible cause-effect relationships among diabetes-related tweets. This task was challenging owing to addressing both explicit and implicit causality, multiword entities, the fact that a word could be both cause or effect, the open domain of causes and effects, the biases occurring during labeling of causality, and the relatively small data set for this complex task. We overcame these challenges by augmenting the small data set via an active learning loop. The feasibility of our approach was demonstrated using modern BERT-based architectures in the preprocessing and causal sentence detection. A combination of BERT features and CRF layer were leveraged to extract causes and effects in diabetes-related tweets, which were then aggregated to clusters in a semisupervised approach. The visualization of the cause-effect network based on Twitter data can deepen our understanding of diabetes, in a way of directly capturing patient-reported outcomes from a causal perspective. The fear of death owing to the inability to afford insulin was the main concern expressed.

## Appendix

Multimedia Appendix 1 List of diabetes-related keywords for the Twitter application programming interface tweet extraction.

Multimedia Appendix 2 Preprocessing pipeline.

Multimedia Appendix 3 Annotation guidelines.

Multimedia Appendix 4 Most frequent clusters.

## Abbreviations

BERT: Bidirectional Encoder Representations from Transformers
BIO: Beginning, Inside, Outside
CRF: conditional random field
