# Supplementary material for: Extraction of Explicit and Implicit Cause-Effect Relationships in Patient-Reported Diabetes-Related Tweets From 2017 to 2021: Deep Learning Approach
Source: JMIR Med Inform. 2022 Jul 19;10(7):e37201. doi: 10.2196/37201 (PMC9346561; doi:10.2196/37201)
Supplement: Multimedia Appendix 1 [file medinform_v10i7e37201_app1.pdf]

Multimedia Appendix 1: List of diabetes-related keywords for the Twitter API tweet extraction

English keywords		
glucose	insulin	blood glucose
#glucose	#insulin	#bloodglucose
insulin pump	diabetes	t1d
#insulinpump	#diabetes	#t1d
type 1	t2d	type 2
#type1	#t2d	#type2
#bloodsugar	#dsma	#type2diabetes
#doc	#bgnow	#wearenotwaiting
#insulin4all	dblog	diyps
hba1c	#dblog	#diyps
#hba1c	#cgm	#freestylelibre
diabetic	#gbdoc	freestyle libre
#diabetic	#gdm	finger prick
gestational diabetes	continuous glucose monitoring	#fingerprick
#gestational	#continuousglucosemonitoring	#changingdiabetes
#thisisdiabetes	#lifewithdiabetes	#diabetesadvocate
#stopdiabetes	#diabadass	#diabetesawareness
#diabeticproblems	#justdiabeticthings	#t1dlookslikeme
#diaversary	#diabetestest	#t2dlookslikeme
pwd	#duckfiabetes	#GBDoc
#pwd	#kissmyassdiabetes	

S1: List of diabetes-related keywords for the Twitter API tweet extraction
