# Supplementary material for: Extraction of Explicit and Implicit Cause-Effect Relationships in Patient-Reported Diabetes-Related Tweets From 2017 to 2021: Deep Learning Approach
Source: JMIR Med Inform. 2022 Jul 19;10(7):e37201. doi: 10.2196/37201 (PMC9346561; doi:10.2196/37201)
Supplement: Multimedia Appendix 2 [file medinform_v10i7e37201_app2.pdf]

Multimedia Appendix 2: Preprocessing pipeline

Tweets are noisy, unstructured and contain numerous misspelled or non-standard english words. To reduce noise in the dataset a similar preprocessing pipeline as in earlier works was adopted [9]. First, retweets and duplicates were removed to obtain a database with 7.7 million unique tweets. Secondly, to increase the relevance of the analyzed tweets we determined only tweets with *personal* content where feelings, emotions and opinions could be shared by people with or talking about diabetes. As a consequence, *institutional* tweets referring to commercial, news or health information, were considered out of scope for this study and excluded. It has already been shown that diabetes-related tweets can be grouped into several clusters such as commercial, health information, social intervention by Beguerisse-Diaz et al [37]. Contrary to Johnsen et al [38] who identified personal content based on personal pronouns like 'I', 'me', 'us', we leveraged the transfer learning paradigm and fine-tuned an already pretrained transformer-based language model to detect personal content in tweets [25]. As pretrained language model served *BERTweet* [32], which was trained on 850 million english tweets (16 billion word tokens ~ 80GB) collected from january 2012 to august 2019 following the RoBERTa pretraining procedure [39]. We undertook the same preprocessing steps to our tweets that were used to pretrain the model such as tokenization, translating emotion icons into text strings and converted user mentions and web/url links into special tokens. To use the model and fine-tune it for a binary sentence classification a linear layer was added on top of the last Transformer layer of the *Bertweet* model using the *transformers* package of Huggingface [33]. The model was then fine-tuned with an extended data set, of the one provided by Ahne et al leading to a total of 4303 tweets (1539 *personal*, 2764 *institutional*), to account for a possible temporal divergence of the way people tweet [9]. The model performance to identify tweets with personal content was: accuracy of 91,2%, f1 of 88,5%, precision of 86,2% and recall of 90,9%. The trained model is then applied on all unique tweets resulting in a total of 2.5 million tweets with personal content.

Moreover, jokes around diabetes are common on Twitter and considered out of scope for this study as well. Similarly to the *personal* content classifier, *BERTweet* was fine-tuned to detect if a tweet is a joke. For this purpose a joke tweet dataset from earlier works was extended to a total of 1648 tweets (486 jokes, 1162 non jokes) [9]. The performance to identify if a tweet is a joke was: accuracy of 90,4%; f1 of 84,2%, precision of 78,5%, recall of 90,8%. Applying the joke classifier on all tweets with personal content led to a dataset of 1.8 million personal, non-joke tweets.

A particular focus of this study lied on studying diabetes distress and thus psychological factors and emotions. To capture those psychological factors and emotions in tweets, only tweets containing an emotional element such as emojis and emoticons or emotional words were kept, analogous to earlier works [9]. In addition, emotional words were identified based on a combination of the psychologue

Parrot's hierarchical classification of emotions with the six primary emotions (*joy, love, surprise, sadness, anger, fear*) and emotional words present in common questionnaires to study diabetes distress such as the Problem Areas in Diabetes scale (PAID) and Diabetes Distress Scale (DDS) [34-36]. Those emotional words were augmented by synonyms using the WordNet database to obtain a more extensive list [40]. This led to 562,013 tweets containing personal, non-joke and emotional content.
