# Supplementary material for: Extraction of Explicit and Implicit Cause-Effect Relationships in Patient-Reported Diabetes-Related Tweets From 2017 to 2021: Deep Learning Approach
Source: JMIR Med Inform. 2022 Jul 19;10(7):e37201. doi: 10.2196/37201 (PMC9346561; doi:10.2196/37201)
Supplement: Multimedia Appendix 3 [file medinform_v10i7e37201_app3.pdf]

Multimedia Appendix 3: Annotation guidelines

Objective

The aim of this labeled corpus is to provide a training data set for detecting possible cause-effect-pairs in diabetes and diabetes distress related sentences from Twitter. Diabetes distress regroups all psychological factors related to the day-to-day disease management such as emotional burden, stress, anxiety, emotions, etc.

Data

Between April 2017 and January 2021 diabetes related tweets have been extracted using the Twitter API based on list of diabetes-related keywords, such as "diabetes", "insulin", "hypoglycemia", "#T1D", "#DSMA", "Type 2", "#diabeteslookslikeme", compare Supplementary File 1 for the full list. Based on the extracted tweets a random subsample of 5000 tweets has been selected, and split into sentences, for annotation purposes.

File structure - Columns:

- **Text [String]**: sentence of tweet
- **Intent [String]**: Intent of the tweet. If several intents, they are separated by a semicolon (";") Can take the following values:
    - q: Question in tweet
    - neg: A negation which negating the meaning of the cause or effect in a sentence
    - joke: A joke, an irony or sarcasm is in the tweet

- **Cause [String]:** Words describing the causes. A cause can be composed of several words. If several causes occur in a tweet then they are separated by a semicolon (";")
- **Effect [String]:** Words describing the effect. An effect can be composed of several words. If several effects occur in a tweet then they are separated by a semicolon (";")
- **Causal association [0,1]**: Binary variable of a cause-effect pair occurs in a tweet where 0 means no cause-effect pair and 1 means there is a cause-effect pair

Definition of a cause-effect relationship and annotation rules

The following tweet examples are fictive to ensure privacy.

Non-diabetes or diabetes distress related relationships

The focus on this corpus lies on cause-effect relationships related to diabetes and diabetes distress. For this reason sentences like the following are not labeled as causal. The possible cause here might be "flu" and the effect "die", but "flu" is out of scope in this project.

**Text**	**Intent**	**Cause**	**Effect**	**Causal associatio n**
Scary, i have a 13 year old diabetic daughter however i read 4 thousand or more people a year die in UK just from flu, so why this fuss & panic over corona .				0
Schools are closed to prevent passing the virus , yet ALL DAY LONG they are in the store with parents , putting me and MY HEALTH at risk !				0

S2: Non-diabetes or diabetes distress related relationships

In the second example "heart disease" is not labeled as the *cause* as it is out of scope.

Examples for possible causal associations

**Text**	**Intent**	**Cause**	**Effect**	**Causal association**
Diabetes causes me to have mood swings. :/		Diabetes	mood swings	1
Years of diabetes and all I got is a Spidey-sense like ability to notice any abnormal sensations in my body and about 7 new kinds of anxiety I didn't know existed before I got diagnosed , it sucks , but I'm much stronger because of it .		diabetes	abnormal sensations in my body;anxiety	1
When I am in need of more insulin my body alarms are all going off , making me tired , headache , blurred vision .		in need of more insulin	tired;headache; blurred vision	1
After 10 years of injections and finger pricks, I have finally gotten an Insulin pump and glucose monitor, finally I can start to manage my diabetes even better and improve my health		Insulin pump;glu cose monitor	manage my diabetes even better;improve my health	1

S3: Possible causal associations

The above examples also show that several causes can lead to an effect, and inversely also one cause can lead to several effects.

Implicit relations

The cause-effect relationship is not stated by a *causal link* word

Text	Intent	Cause	Effect	Causal associatio n
I was sent to the penalty box to fix a low blood sugar #diabetes #NHLPlayoffs		#diabetes	low blood sugar	1

S4: Implicit relations

Unclear cause - effect relationships

In sentences in which there is a possible cause and a possible effect but it is not clear if the "cause" had an influence on the "effect", the tweet is labeled as non-causal.

Text	Intent	Cause	Effect	Causal association
Had two strokes recovering now my legs do not want to move and I have high blood pressure and diabetes				0
My dad has diabetes, cancer , heart problems , and a weak immune system .				0

S5: Unclear cause-effect relationships

The possible cause is "High blood sugar and diabetes" and the possible effect is "stroke". But it can not be concluded that the stroke was provoked by the high blood sugar or diabetes

Several chaining cause-effect relationships: A -> B -> C

If in a sentence we have two relationships: event A causes event B and at the same time event B causes event C, then we labelled the relationship that is closest to our objective to study diabetes and diabetes distress:

Text	Intent	Cause	Effect	Causal associatio n
Not sure if I've been up since 3:30 for Titan or because my anxiety over my glucose test is keeping me up 😬 Bahh		glucose test	anxiety	1
I am also a diabetic with all this worry & stress , is adding to my sugar		diabetic	worry;stress	1

levels to rise ..				
Excess insulin from eating too many carbs spikes insulin , making you hungry		eating too many carbs	Excess insulin;spikes insulin	1

S6: Chaining cause-effect relationships

event A: glucose test
event B: anxiety
event C: been up since 3:30

Negations

If a negation word occurs in a cause or effect, it is considered being part of the cause and effect and so not altering the meaning of the cause or effect. Consequently the sentence is not labeled as having a negation “neg” in the Intent.

Text	Intent	Cause	Effect	Causal association
My 14 year old daughter has Type 1 (malfunctioning pancreas , aka not enough insulin being made to regulate		Type 1	malfunctioning pancreas; not enough insulin	1
I'm a Type 1 Diabetic , out of work and unable to afford my insulin 😪		out of work	unable to afford my insulin	1
I was wondering why i felt like shit and then I realized I haven't given myself my insulin since early this morning . stupid..	neg	insulin	felt like shit	1
Don't hate your diabetes ; instead , find ways to love it and get rid of it over time . it helps	neg	diabetes	hate	1
My friend " gave " herself diabetes by not doing what her doctor told her LOSE WEIGHT !	neg	LOSE WEIGHT	diabetes	1

S7: Negation examples

The last example shows when a tweet is labeled as negation. The negation “haven’t given myself” alters the meaning of the causal relationship “insulin” -> “felt like shit”.

Diabetes Distress

In labeling this data set, a special focus was lying on diabetes distress (psychological factors related to the day-to-day disease management, such as anxiety, stress, emotions, etc.). For this reason we labeled possible causal associations related to diabetes distress as well:

Text	Intent	Cause	Effect	Causal association
I do I just want to go to the kitchen and eat, I hate #diabetes		#diabetes	hate	1
I have gestational diabetes and im very much bothered		gestational diabetes	bothered	1
Kent ' s just angry because his diabetes is flaring up again .		diabetes	angry	1

S8: Diabetes distress examples

Jokes

As jokes were also labeled sentences containing ironic or sarcastic elements.

Text	Intent	Cause	Effect	Causal association
This tweet is so dumb it gave me diabetes	joke			0
I love lifestyle choices become a non smoker and a temporary diabetic , why the hell not *irony out*	joke			0
Thanks sweetie And I think I've developed diabetes from your sweetness	joke			0

S9: Joke examples

Frequently used abbreviations related to diabetes

Abbreviation	Explanation
lows, going low	low blood sugar
#gbdoc, #doc, #dsma	diabetes related online groups on social media to exchange about the disease
dexcom, Freestyle Libre	continuous glucose monitoring tools helping to monitor blood sugar levels levels
cgm, CGM	continuous glucose monitoring
DKA, dka	Diabetic Ketoacidosis
3 hours	3 hour glucose test for gestational diabetes
LCHF	low carb high fat diet: The diet, because of its low requirement for insulin, has been recognised by the Swedish government as being suitable for people with type 2 diabetes and as helpful to individuals looking to lose weight or maintain a healthy weight.
BS	blood sugar

S10: Frequently used abbreviations related to diabetes
