# Supplementary material for: Extraction of Explicit and Implicit Cause-Effect Relationships in Patient-Reported Diabetes-Related Tweets From 2017 to 2021: Deep Learning Approach
Source: JMIR Med Inform. 2022 Jul 19;10(7):e37201. doi: 10.2196/37201 (PMC9346561; doi:10.2196/37201)
Supplement: Multimedia Appendix 4 [file medinform_v10i7e37201_app4.pdf]

Multimedia Appendix 4: Most frequent clusters

The synonyms were manually added for the initial clusters. Clusters whose parent cluster is "Other", are automatically added clusters that were predicted from the cause-effect classifier.

Most frequent clusters

N°	Parent cluster	cluster	synonyms	N
0	Diabetes	diabetes	diabetic, #diabetic, #diabetes, diabetes mellitus, diabetics, DIABETIC, #Diabetic	66,775
1	Death	death	die, passed away, gave life, kill, killing, dead, shorter lifespan, loose live, died, dying, commit suicide, losing father	16,989
2	Insulin	insulin	insulin hormone, supplies, HUMALOG bottle	14,148
3	Diabetes	type 1 diabetes	T1D, type 1, #type1, #type1diabetes, juvenile diabetes	11,693
4	Emotions	fear	anxiety, terrified, scared, anxious, concern, dread, scary, worried, nervous, distress, panic, hysteria, terrified, creeped, traumatized	10,160
5	Glycemic variability	hypoglycemia	low glucose, low blood sugar, low, hypo, go low, glucose down, blood sugar down, lowered BS, sugar drop	9547
6	Symptoms	sick	headache, dizzy, threw up, vomiting, painful, puke, feeling sick, shaky, nausea, coughing, diarrhea, dry mouth, fever, sweat	6549
7	Nutrition	overweight	obese, eat too much, weight gain, fat, gained pounds, obesity	5186
8	Diabetes	type 2 diabetes	T2D, type 2, #type2, #type2diabetes, T2 diabetic, t2d, TYPE TWO	4909
9	Complications & comorbidities	neuropathy	amputation, feet amputation, lost feet, lost leg, leg amputation, nerve death, nerve damage, lost hand, diabetes neuropathy	4481
10	Health care system	medication	meds, diabetes meds, drug, antibiotics, pills, drugs, dose, medicine, metformin prescribed, prescription, cheapest medicines	4389
11	Diabetes Technology	insulin pump	injecting insulin, injection, inject, needle, pump, finger prick, shot,	4307
12	Nutrition	nutrition	vegan, vegetarian, eat carbs, carbohydrates, no chocolate, can't eat donuts, food, salad, noodles, appetite fish, NEEDED meal, seafood, milk, entire meal, broccoli	4230
13	Emotions	anger	rage, outrageous, frustration, hate, angry, jealous, jealousy, raging, pissed, pissed off, frustrating	4149
14	Health	OGTT	glucose test drink, glucose test, 3 hour test, ogtt, diabetic drink, horrific drink	4053
15	Blood pressure	hypertension	high blood pressure, BP	3782
16	Health care system	finance	wages, student loans, GoFundMe, expenses, costly, expensive care, pay, price gouging, spend money, debt, insulin price, insulin prices, donations, Healthcare Cost	3767
17	Nutrition	reduce weight	lost pounds, lose weight, #loseweight	3589
18	Insulin	unable to afford insulin	can't afford insulin, no access to affordable insulin, could not afford insulin, can't afford meds, could not buy insulin, bankrupt, financially unstable	3381
19	Nutrition	diet	diabetic diet, Keto diet, carnivore diet, keto, plant based diet, high fat diet, change diet, #lowcarb, LCHF, dietary needs, Low Carb	3325
20	Emotions	sadness	cry, sad, sucks, loneliness, lonely, sadly, CRIED, despair, hurtful, hurting, psychological grief, disappointing	3153

21	Glycemic variability	hyperglycaemia	high blood sugar, high glucose, high glucose levels, spike glucose, higher blood glucose, blood glucose up, blood glucose levels up, elevated #BP, rebellious #hyperglycemia	3144
22	Diabetes	suffer	suffering, TERRIBLE PAIN, HURT	3132
23	Diabetes Distress	depression	depressed, depressing, lose hope, mentally ill, hopeless, antidepressants, psychologically fragile	2810
24	Health care system	hospital	surgery, syringes, doctor, appointment, checkup, medical attention, medical treatment, ER, ICU, hospitalize, ambulance, doc, surgeries, GP practice, clinical psychologist, Caregiver	2721
25	Diabetes Distress	stress	mood disorder, stressed, stressful,, MOOD SWINGS, tense	2681
26	Nutrition	sugar	sweets, candy, waffle, soda, Sugar, CAKE, artifical sweeteners, CRAVE SWEETS, milkshakes, LOVE SUGAR	2369
27	Nutrition	fasting	starvation, not eating	2363
28	Insulin	rationing insulin	shortage insulin, denying insulin, lack insulin, ration insulin, EXPIRED INSULIN	2244
29	Health	gestational diabetes	pregnancy, pregnant	2076
30	Health	prediabetes	pre diabetic, borderline diabetic	1932
31	Diabetes Distress	feel bad	feel awkwared, disgusting, appetite, grumpy	1861
32	Complications & comorbidities	retinopathy	horrible vision, bad eyesight, vision decline, lost sight, blind, diabetes retinopathy	1750
33	Complications & comorbidities	high risk	risk	1663
34	Health care system	insurance	company, pharma, health insurance, coverage, Medicare, #BigPharma #Insulin, medicaid	1627
35	Complications & comorbidities	coma	unconscious, pass out, Diabetic Coma	1540
36	Complications & comorbidities	heart attack	cardiovascular, cardiovascular disease, diabetic heart attack, CHF	1511
37	Health	insulin resistance		1505
38	Complications & comorbidities	complications	diabetes complication, issues	1443
39	Other	struggle		1357
40	Complications & comorbidities	nephropathy	kidney damage, diabetes kidney failure, Nephrologist #Diabetes #Nephrologist,kidney failure	1338
41	Emotions	joy	feel good, feel better, relief, happy, proud	1279
42	Other	constant pain		1240
43	Other	n't know language		1234
44	Diabetes Distress	fatigue	no power, without energy, exhaused, tired, lethargic, burnout, exhaustion	1190
45	Pandemic	covid	corona, coronavirus, virus, covid pandemic business, Corona, vaccine, worrying COVID, severity COVID, VIRUS, #CoronavirusPandemic, SARS CoV 2 INFECTION, vaccinated	1073
46	Lifestyle	physical activity	exercising, walking, sport, exercises, walk, gym, gyms	1066
47	Health care system	politics	health system, NHS, #brexit, brexit, Canada, capitalism, government, EU, administration, frustrated #NHS, economy, CANADA, CAPITALISM	985
48	Insulin	access insulin	no insulin, don't have insulin, without insulin, #insulin4all, #Insulin4all #Diaversary	956
49	Diabetes Technology	continuous glucose monitor	freestyle libre, #freestylelibre, monitoring, #dexcom, CGM, cgm	934
50	Insulin	affordable insulin	afford insulin	915
51	Complications &	shock	dIaBeTiC sHOcK	908

	comorbidities			
52	Pandemic	home	staying home, quarantine, shutdown	864
53	Diabetes	management	control diabetes, uncontrol	825
54	Complications & comorbidities	infection	wound, wounds, inflammation	822
55	Symptoms	Insomnia	can't sleep, awake, wake, sleepy, asleep	737
56	Health	lost job	without work, laid off	723
57	Complications & comorbidities	diabetic ketoacidosis	keto acidosis, #ketoacidosis, diabetic ketoacidosis, DKA, #ketoacidosis, keto acidosis, DAIBETIC KETO ACIDS high, #KETO #NSNG	710
58	Health	immune system		705
59	Nutrition	eating healthy	#healthy #meal	608
60	Blood pressure	hypotension	low blood pressure	587
61	Family	family	brother, daughter, grandpa, dad, mom, grandma, parent	581
62	Complications & comorbidities	renal failure	diabetes renal failure, dialysis	554
63	Complications & comorbidities	legs swollen	foot swelled	544
64	Diabetes	reverse diabetes	reversed, cured overnight	489
65	Diabetes community	support	#dsma, help, raise awareness, supporters	483
66	Lifestyle	lifestyle	environment	453
67	Emotions	love	like	451
68	Other	cold		443
69	Complications & comorbidities	cholesterol		392
70	Other	meat fake meat		376
71	Other	starve		363
72	Diabetes Distress	isolation	alone, live alone, distrust	358
73	Other	cut back rice		348
74	Other	taking necessary precautions		346
75	Complications & comorbidities	pancreas	diabetes pancreas	340
76	Health	PCOS	pcos, PCOs, Pcos	333
77	Other	ass alive		318
78	Other	arthritic		312
79	Other	dangerous		301
80	Other	vulnerable		299
81	Other	seizures		285
82	Other	acting		274
83	Other	needles sensation		273
84	Other	slightly concerned		267
85	Insulin	insulin spike	insulin jump	263
86	Complications & comorbidities	liver failure	diabetes liver failure	256
87	Other	fucked drunk		253
88	Complications & comorbidities	stomach		246

89	Symptoms	thirsty	thirst, dehydrated, THIRSTY	245
90	Other	stop talking		244
91	Nutrition	alcohol	beer, BEER, alcoholism	243
92	Glycemic variability	A1C	a1c, predict HbA 1c	236
93	Health	genetic	genes, Genetics, hereditary, genetically modified, shit genetics	228
94	Complications & comorbidities	cancer	chemo, #Cancer	226
95	Other	hair fall		226
96	Other	crash hard		212
97	Other	shut kidneys		211
98	Other	isolated ' society		210
99	Other	afraid ingredients		204
100	Other	surgeon excited		202

S11: Top 100 most frequent clusters
